# Primary intradural extramedullary Ewing sarcoma in the lumbar area: A case report

**DOI:** 10.1016/j.radcr.2022.09.033

**Published:** 2022-09-29

**Authors:** Ramin Ebrahimi, Amir sajjad Mounesi sohi, Ali Mirsardoo, Nima Moosavi, Mohammad Saeid Khonji

**Affiliations:** a Department of Radiology, Firoozgar Hospital, Iran University of Medical Sciences, Tehran, Iran; b Department of Radiology, Iran University of Medical Sciences, Tehran, Iran; c Bone and Joint Reconstruction Research Center, Iran University of Medical Sciences, Tehran, Iran

**Keywords:** Ewing sarcoma, Spinal cord neoplasms, Intradural extramedullary, Tumors, Spinal cord

## Abstract

Primary intradural extramedullary Ewing sarcoma (IEES) is the rarest type of Ewing sarcoma. Extreme caution is required for the diagnosis of IEES because benign intradural spinal tumors can be mistaken for IEES in the early stages of imaging and clinical evaluation. IEES tumors have no standardized treatment guidelines because of the lack of research on the therapeutic aspects of these tumors. Herein, we present a case of primary IEES in a male adolescent with a fast progression of the disease. Diagnosis of IEES was suspected with imaging (computed tomography scan and lumbosacral magnetic resonance imaging) and was confirmed with histology and immunohistochemistry (positive reactivity for CD99 and FLI1). He was successfully treated with surgical intervention with no radiotherapy or chemotherapy. Imaging studies are helpful in making the initial diagnosis of intradural extramedullary Ewing sarcoma. Surgery is considered to be a successful method of treatment for this condition.

## Introduction

Tumors arising from the spinal cord/cauda equina are rare entities and include a wide range of diagnoses. Meningioma, nerve sheath tumors, such as schwannoma or neurofibroma, astrocytoma, ependymoma, spinal involvement of extraskeletal of metastatic disease and Ewing sarcoma in the epidural space or paravertebral area are the primary diagnoses for intradural spinal tumors [1]. However, the majority of spinal cord tumors extramedullary entities [1,2]. Ewing sarcoma is part of a group of high-grade small round cell tumors that also includes pPNET and Askin tumor. It is an aggressive tumor of bone and soft tissue that is more common in adolescents and young adults [3]. Ewing sarcoma is often detected in the long bones, pelvis, or ribs. It can also rarely arise in extraskeletal locations such as the paravertebral or epidural space.

However, primary intradural extramedullary Ewing sarcoma (IEES) is the rarest kind of Ewing sarcoma [4]. Due to the fact that the first imaging and clinical findings of IEES can mimic those of benign intradural spinal tumors, extreme consideration is essential when diagnosing IEES. Consequently, oncologists and neurosurgeons should be accustomed to the presentation and assessment of IEES. Moreover, IEES is an aggressive malignant tumor that leads to severe neurologic morbidity and mortality without treatment. Nonetheless, due to the extremely low prevalence, there isn't much known about how to treat IEES, so there aren't any standard guidelines for how to treat these tumors. Even so, the current treatments for IEES include surgery, focal radiotherapy, and systematic chemotherapy [3]. Herein, we present a 13-year-old boy with a sudden inability to walk that was diagnosed with IEES.

## Case presentation

A 13-year-old boy with no previous medical history of note appeared with a sudden inability to walk that began 24 hours prior. He had developed severe pain in the left lower extremity for a week ago. His symptoms were progressive; however, he had no motor problem till 24 hours ago. On physical examination, the upper extremities were normal. Bladder and bowel functions were also normal. Babinski's reflexes were negative on both sides. In addition, on both sides, the straight leg raise and femoral nerve stretch tests were negative.

**Fig. 1:**
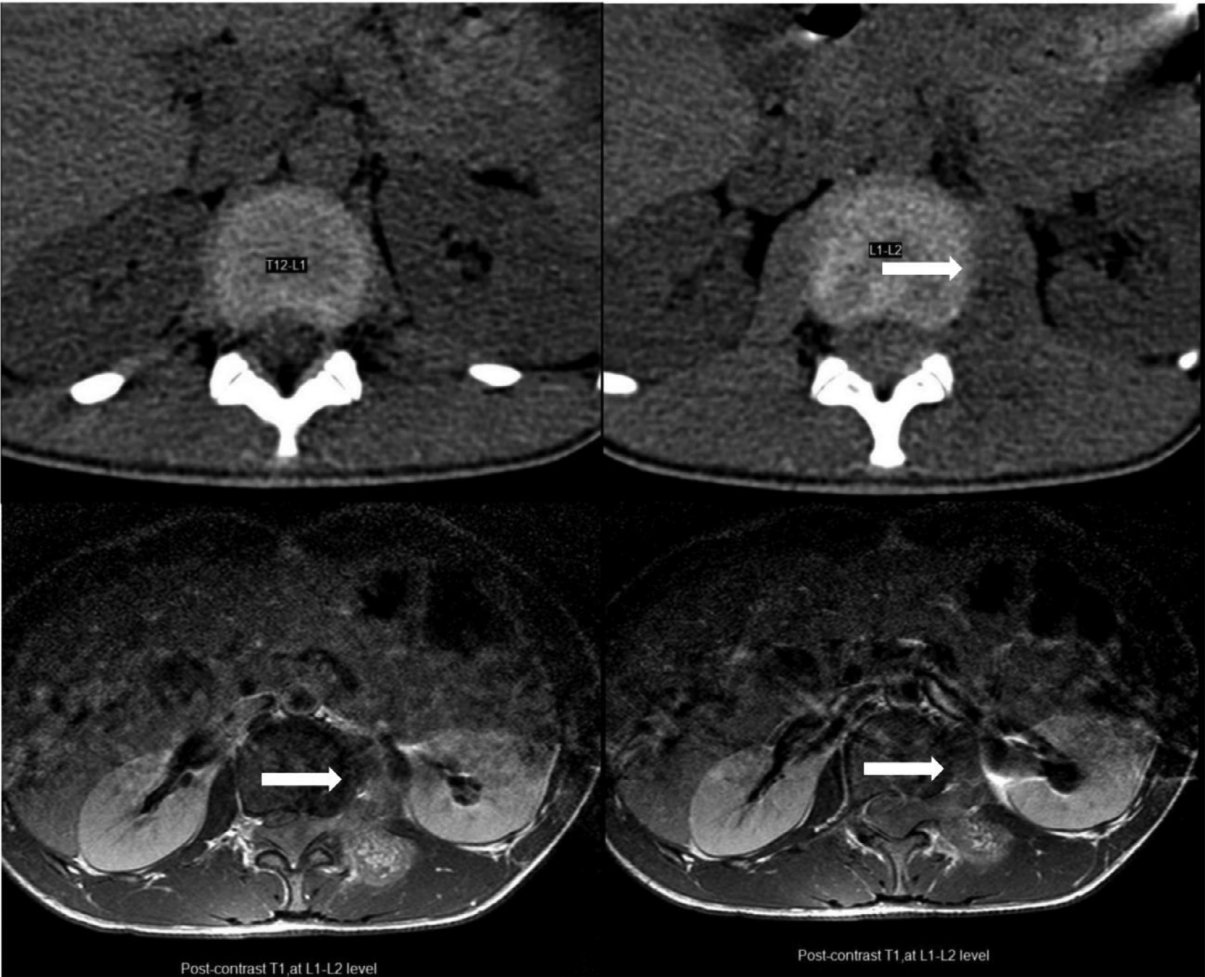
Computed tomography (CT) scan of the thoracolumbar spine shows s mass-like lesion with soft tissue density was seen on the left side of the spinal canal at the L1-L2 level with extension to the neural foramen and the left paraspinal muscles at the same level. White arrows indicate mass lesion.

**Fig. 2:**
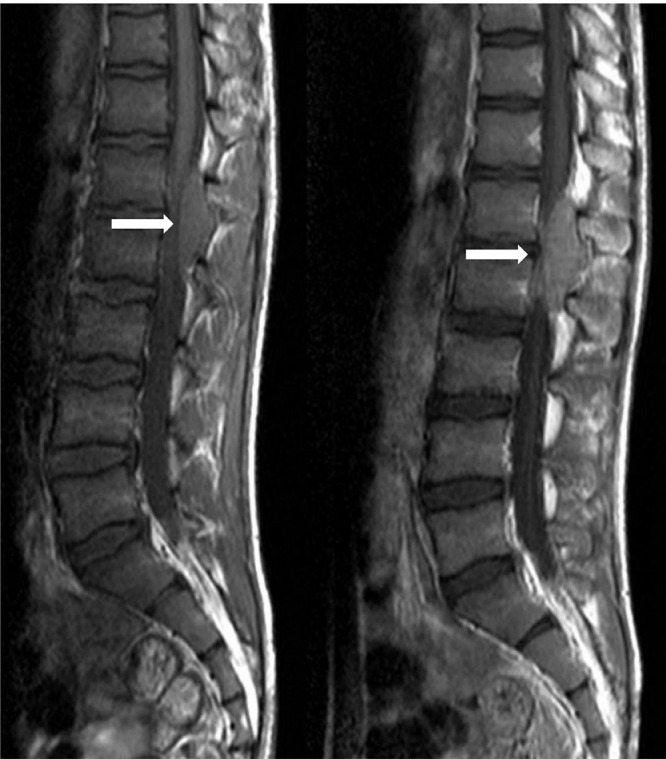
lumbosacral magnetic resonance imaging (MRI) without and with gadolinium revealed an intradural extramedullary mass in the spinal canal at L1-L2 level. White arrows indicate mass lesion.

**Fig. 3:**
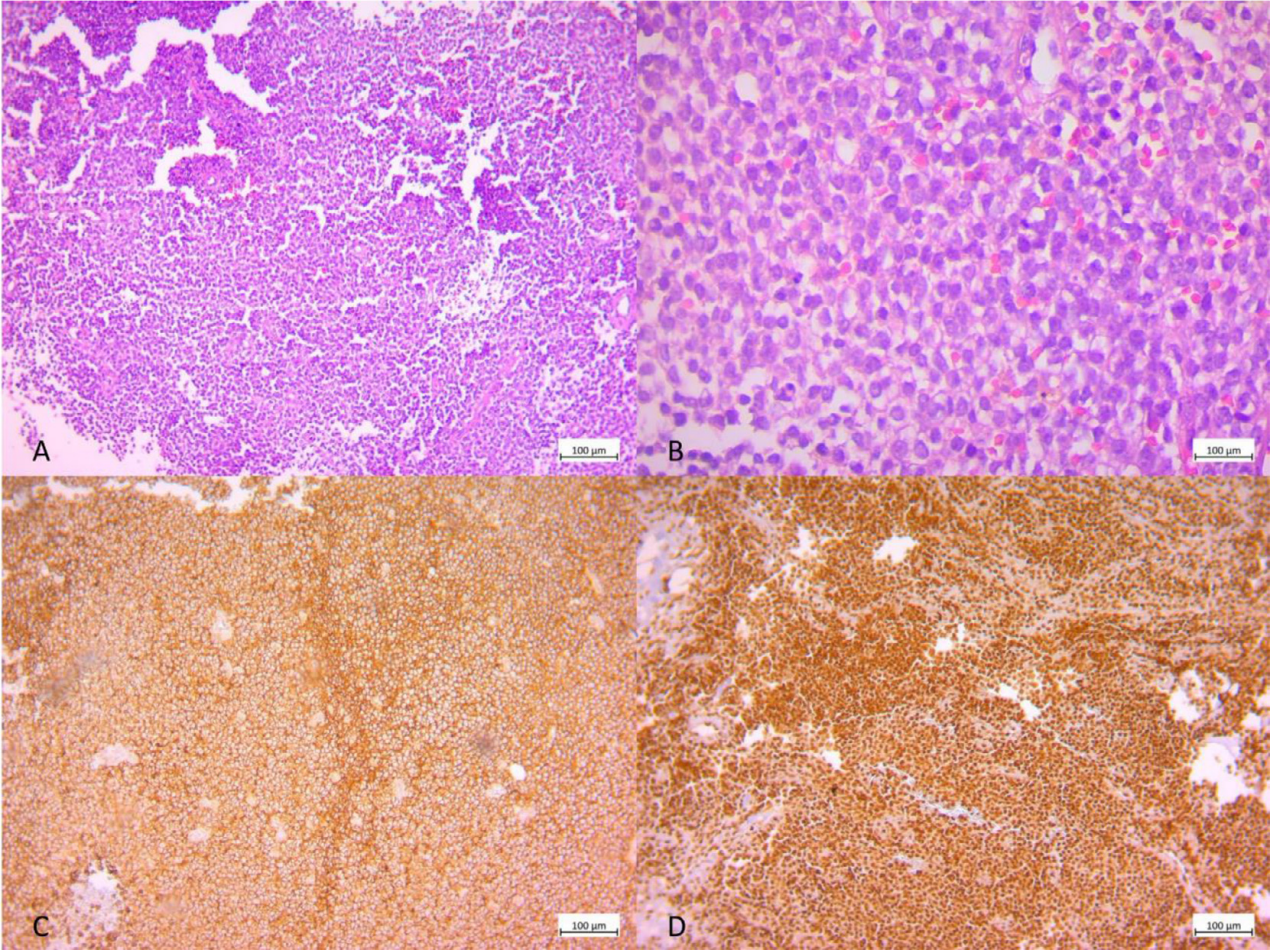
Histopathology images. (A) Hematoxylin and Eosin staining with fourth times magnification (×4). (B) Hematoxylin and Eosin staining with 40th times magnification (×40). (C) IHC staining for CD99 (positive). (D) IHC staining for FLI1 (positive).

The patient was assessed with a spiral computed tomography (CT) scan of the thoracolumbar spine (Fig. 1); Epidural fat was obliterated at the L1-L2 level. A mass-like lesion with soft tissue density was seen on the left side of the spinal canal at the L1-L2 level with extension to the neural foramen and the left paraspinal muscles at the same level. No evidence of osseous remodeling or destruction was noted. A subsequent lumbosacral magnetic resonance imaging (MRI) without and with Gadolinium revealed an intradural extramedullary mass in the spinal canal at L1-L2 level (Fig. 2). The mass displayed iso-signal intensity on T1 imaging and mild heterogeneous greater signal intensity on T2-weighted images in comparison to paraspinal muscles and had caused spinal cord compression and right anterolateral displacement. The mass showed homogeneous enhancement after contrast injection. Evidence of extension to the neural foramen was seen on the left side with involvement of the left psoas and left posterior paraspinal muscles. Multiple spinal cord samples were sent for pathology and IHC evaluations. Microscopic evaluation showed sheets of neoplastic cells with oval to round hyperchromic nuclei in fibrotic stroma, with pieces of trabecular bone (Figs. 3A and B). Immunohistochemistry (IHC) also showed positive reactivity for CD99 and FLI1 and negative reactivity for LCA (Figs. 3C and D). After the diagnosis of Ewing sarcoma, the patient was scheduled for surgical treatment. After midline incision, laminectomy of L1 and L2 and removal of L2 transverse process was performed, and the lesion was totally removed. Moreover, the remaining tumor in the paravertebral muscles was also removed.

## Discussion

We present an extremely rare case of lumbar IEES in an adolescent male. Reviewing the literature, only less than 50 cases of IEES has been reported in the cervical, thoracic and lumbosacral area. In a recent systematic review by Lu et al. [5], 44 cases of IEES were found. The median age of diagnosis of IEES was 31 years, with 70% male incidence. Our case was male, but his age was lower than the average age. Moreover, the lumbar/sacral region was the most common location (61%) as in our study. The most common chief complaints have been pain and weakness as in our case. The symptom duration of our case was one week, while the average duration of the chief symptom in other cases was 3 months. We treated the patient with surgical intervention. In agreement, all previous cases have been treated with surgical intervention, followed by adjuvant chemotherapy and local radiation therapy [5].

IEES was diagnosed by pathological investigation in our research, which revealed positive reactivity for CD99 and FLI1. Histological assessment is the main evaluation for diagnosis. Previous research has shown that tiny, spherical, blue cells accompanied by positive CD99 antigen expression are suggestive of Ewing sarcoma. CD99 is a cell membrane glycoprotein that might inhibit cellular differentiation by suppressing the MAPK pathway in Ewing sarcoma [6]. Regardless, CD99 expression is not unique to Ewing sarcoma. It is also found in other types of primitive neuroectodermal cancers. A more specific diagnosis of primary IEES can be achieved with a positive molecular EWSR1-FLI1 fusion transcript [7]. Like our case, the diagnosis of most cases in the literature was based only on histopathology and IHC. However, it should be noted that histopathology and IHC are not 100% specific for IEES. Another diagnosis that is extremely similar to IEES is peripheral PNET. These tumors also are positive for EWSR1-FLI1 fusion transcript and CD99. Several cases in the literature have therefore described their findings as “Ewing sarcoma/PNET”. Similarly, we also could not discriminate between these 2 types of tumors in our case.

Although the pathogenesis of Ewing sarcoma of the spine is ambiguous, initial evidence suggests that the cell of origin is responsible for tumor development. In fact, based on the expression of neuroectodermal markers on tumor cells, it was postulated that Ewing sarcoma originates from neural crest cells [5]. Nonetheless, new evidence shows that production of the EWSR1-FL11 fusion transcript increases expression of neural crest-related genes in bone marrow cells and fibroblasts [8].

Surgical intervention is the mainstay of treatment of spinal Ewing sarcoma. Previous studies reported that complete resection with negative margins of extraosseous Ewing sarcoma leads to increased survival [5]. However, we did not report the survival of our patient in this study. There is less evidence regarding the efficacy of adjuvant treatment, including radiotherapy and chemotherapy. Saeedinia et al. [9] conducted a meta-analysis on extradural Ewing sarcoma and observed that treatment with both modalities leads to increased survival at 1-year follow-up compared to the situation where one modality is applied. Nonetheless, there is less evidence regarding primary IEES.

In conclusion, radiographic assessments play a key part in the primary diagnosis of IEES and surgery is regarded to be a successful means of treatment. Further evaluations in the future may be helpful.

## Author contributions

R. E., A. M., A. S., N. M. and M. S. K. conceived and designed the evaluation. R. E., A. M., A. S., N. M. and M. S. K. helped to collect clinical data, draft the manuscript and revised the manuscript.

## Data availability

The data that support the findings of this study are available from the corresponding author, upon reasonable request.

## Ethics approval

This study is approved under the ethical approval. (webpage of ethical approval code is:https://ethics.research.ac.ir/PortalCommittee.php?code=IR.IUMS.FMD.REC)

## Patient consent

Informed consent for participation in the study and publication was obtained from the patient's father.
